# Supplementary material for: The Effectiveness of Artificial Intelligence in Assisting Mothers with Assessing Infant Stool Consistency in a Breastfeeding Cohort Study in China
Source: Nutrients. 2024 Mar 15;16(6):855. doi: 10.3390/nu16060855 (PMC10975400; doi:10.3390/nu16060855)
Supplement: Supplementary file 1 [file nutrients-16-00855-s001.zip › nutrients-2893034-Supplementary.pdf]

Table S1. Comparison of maternal and infant characteristics between whether or not uploading pictures.

Characteristics		Upload pictures (n = 98)	Non-upload pictures (n = 33)	*p*
Age of mother (years) [1]	< 30	37 (37.8)	9 (27.3)	0.483
	30-35	44 (44.9)	16 (48.5)	
	> 35	17 (17.3)	8 (24.2)	
Occupation [1]	Employed	86 (87.8)	31 (93.9)	0.320
	Unemployed/Housewife	12 (12.2)	2 (6.1)	
Education level [1]	Junior college and below	34 (34.7)	9 (27.3)	0.432
	University and above	64 (65.3)	24 (72.7)	
Per Capita Monthly Income(yuan/month) [1]	< 6250	21 (21.4)	9 (27.3)	0.620
	6250-12500	51 (52.0)	14 (42.4)	
	> 12500	26 (26.6)	10 (30.3)	
Parity [1]	Multiparity	40 (40.8)	11 (33.3)	0.446
	Primiparity	58 (59.2)	22 (66.7)	
Pregnancy Complication History [1]	Yes	60 (61.2)	18 (54.5)	0.499
	No	38 (38.8)	15 (45.5)	
Mode of delivery [1]	Vaginal	65 (66.3)	15 (45.5)	0.033
	Caesarean	33 (33.7)	18 (54.5)	
Postpartum depression [1]	Yes	30 (30.6)	13 (39.4)	0.353
	No	68 (69.4)	20 (60.6)	
EPDS score [2]		7 (4, 12)	7 (4, 10)	0.578
The total score of IGSQ [2]		25.0 (21.0, 31.0)	22.0 (18.0, 27.0)	0.012
Stool characteristics [2]		2.0 (2.0, 2.0)	2.0 (2.0, 2.0)	0.810
Vomiting [2]		6.5 (5.0, 8.0)	7.0 (5.0, 8.0)	0.640
Crying [2]		7.0 (4.0, 9.0)	4.0 (3.0, 6.5)	0.001
Fussiness [2]		4.0 (2.0, 6.0)	2.0 (2.0, 4.0)	0.017
Bloating [2]		6.0 (4.0, 7.0)	5.0 (2.0, 6.0)	0.289
The total score of PedsQL scale [3]		68.9 ± 11.7	74.4 ± 12.1	0.021
Physiological functioning [2]		66.7 (54.2, 87.5)	75.0 (62.5, 100.0)	0.011
Physical symptoms [2]		85.0 (77.5, 90.0)	87.5 (82.5, 90.0)	0.168
Emotional functioning [2]		79.2 (68.2, 91.7)	83.3 (74.0, 93.8)	0.106
Social functioning [2]		50.0 (31.3, 75.0)	62.5 (34.4, 93.8)	0.139
Cognitive functioning [2]		12.5 (6.3, 31.3)	12.5 (0.0, 25.0)	0.346
Psychosocial health summary score [3]		62.0 ± 15.0	67.4 ± 15.3	0.080
Physical health summary score [3]		77.4 ± 11.3	83.2 ± 11.1	0.012

EPDS, Edinburgh Postnatal Depression Scale; IGSQ, Infant Gastrointestinal Symptom Questionnaire.
[1] presented as N (%).
[2] presented as median (P25, P75).
[3] presented as $\bar{x}$ ± SD.

		BITSS Score: Researcher						
		1	2	3	4	5	6	7
BITSS Score: AI	1	0	0	0	0	0	0	0
	2	0	0	0	0	0	0	0
	3	0	0	0	0	1	1	0
	4	0	0	0	0	0	1	0
	5	0	0	0	0	0	0	0
	6	0	0	0	0	4	822	7
	7	0	0	0	0	0	27	42

Figure S1. Agreement between AI and researchers' BITSS scores of 905 stool photographs. The 7 BITSS types are grouped into 4 categories: hard (1-3), formed (4), loose (5-6), and watery (7). BITSS = Brussels Infant and Toddler Stool Scale.

Table S2. Tobit regression analysis of mothers' deviation under different education levels.

Stratified Condition	Characteristics	*β* (95%CI)	SE	*z*	*p*
**Junior college and below**	constant	0.024 (-0.571, 0.620)	0.304	0.080	0.936
	**EPDS score**	0.025 (-0.002, 0.051)	0.014	1.824	0.068
	**Mode of delivery (ref = Vaginal)**				
	Caesarean	0.114 (-0.229, 0.456)	0.175	0.650	0.516
	**Occupation (ref = Employed)**				
	Unemployed/Housewife	-0.042 (-0.411, 0.327)	0.188	-0.223	0.824
**University and above**	constant	-1.269 (-2.238, 0.300)	0.494	-2.566	0.010
	**EPDS score**	0.028 (-0.007, 0.063)	0.018	1.590	0.112
	**Mode of delivery (ref = Vaginal)**				
	Caesarean	0.217 (-0.131, 0.564)	0.177	1.220	0.222
	**Occupation (ref = Employed)**				
	Unemployed/Housewife	0.804 (0.113, 1.496)	0.353	2.281	0.023

EPDS, Edinburgh Postnatal Depression Scale; CI, confidence interval.

Table S3. Tobit regression analysis of mothers' deviation under different delivery modes.

Stratified Condition	Characteristics	*β* (95%CI)	SE	*z*	*p*
**Caesarean**	constant	-0.322 (-1.544, 0.900)	0.623	-0.516	0.606
	**EPDS score**	0.040 (0.025, 0.075)	0.018	2.262	0.024
	**Education level (ref = Junior college and below)**				
	University and above	0.028 (-0.420, 0.477)	0.229	0.124	0.901
	**Occupation (ref = Employed)**				
	Unemployed/Housewife	0.214 (-0.281, 0.709)	0.253	0.849	0.396
**Vaginal**	constant	0.098 (-0.802, 0.999)	0.460	0.214	0.830
	**EPDS score**	0.014 (-0.015, 0.044)	0.015	0.955	0.339
	**Education level (ref = Junior college and below)**				
	University and above	-0.204 (-0.515, 0.107)	0.159	-1.287	0.198
	**Occupation (ref = Employed)**				
	Unemployed/Housewife	0.220 (-0.304, 0.743)	0.267	0.822	0.411

EPDS, Edinburgh Postnatal Depression Scale; CI, confidence interval.

Table S4. Tobit regression analysis of mothers' deviation under different occupations.

Stratified Condition	Characteristics	*β* (95%CI)	SE	*z*	*p*
**Employment**	constant	0.159 (-0.510, 0.827)	0.341	0.464	0.642
	**EPDS score**	0.024 (0.000, 0.048)	0.012	1.960	0.050
	**Education level (ref = Junior college and below)**				
	University and above	-0.255 (-0.535, 0.026)	0.143	-1.779	0.075
	**Mode of delivery (ref = Vaginal)**				
	Caesarean	0.160 (-0.118, 0.439)	0.142	1.127	0.260
**Unemployment/house-wife**	constant	-0.858 (-1.812, 0.097)	0.487	-1.762	0.078
	**EPDS score**	0.036 (-0.011, 0.082)	0.024	1.508	0.132
	**Education level (ref = Junior college and below)**				
	University and above	0.601 (0.163, 1.039)	0.224	2.687	0.007
	**Mode of delivery (ref = Vaginal)**				
	Caesarean	0.187 (-0.239, 0.613)	0.218	0.859	0.390

EPDS, Edinburgh Postnatal Depression Scale; CI, confidence interval.
